# Patterns of failure after resection of extrahepatic bile duct cancer: implications for adjuvant radiotherapy indication and treatment volumes

**DOI:** 10.1186/s13014-018-1024-z

**Published:** 2018-05-08

**Authors:** Hoon Sik Choi, Ki Mun Kang, Bae Kwon Jeong, Hojin Jeong, Yun Hee Lee, In Bong Ha, Tae Gyu Kim, Jin Ho Song

**Affiliations:** 1 Department of Radiation Oncology, Gyeongsang National University School of Medicine and Gyeongsang National University Changwon Hospital, 13 Samjungja-ro, Changwon, 51472 Republic of Korea; 2 Department of Radiation Oncology, Gyeongsang National University School of Medicine, and Gyeongsang National University Hospital, Jinju, Republic of Korea; 3 Institute of Health Science, Gyeongsang National University, Jinju, Republic of Korea; 4 Department of Radiation Oncology, Samsung Changwon Hospital, Sungkyunkwan University School of Medicine, 158 Paryong-ro, Masanhoewon-gu, Changwon, 51353 Republic of Korea

**Keywords:** Bile duct neoplasms, Recurrence, Adjuvant radiotherapy

## Abstract

**Background:**

The role of adjuvant radiotherapy (RT) and setting proper RT target volumes have not been clearly demonstrated for extrahepatic bile duct (EHBD) cancer, due to the rarity of the disease and the lack of randomized trials. This study was conducted to evaluate the indication and treatment volume for adjuvant RT in EHBD cancer patients by identifying the prognostic factors for loco-regional (LR) failure, and analyze the patterns of LR failure.

**Methods:**

Ninety-three patients with EHBD cancer, who underwent resection without adjuvant RT, at 2 medical centers, between 2001 and 2016, were analyzed retrospectively. Univariable and multivariable analyses were performed to find the prognostic factors for LR recurrence. The initial patterns of failure were recorded, especially those of LR recurrence, and categorized according to the Japanese classification.

**Results:**

The median follow-up duration was 30 months, and 38 (40.9%) patients experienced LR recurrence during this period. With regards to LR recurrence, close or positive resection margin (RM) status (*p* < 0.001) remained statistically significant in the multivariable analysis. The most common LR recurrence sites were the tumor bed (18.3%), and lymph node (LN) stations No. 8 (14.1%), No. 9 (12.7%), No. 12 (12.7%), No. 13 (5.6%), No. 14 (21.1%), No. 16 (14.1%), and No. 17 (1.4%).

**Conclusions:**

A close or positive RM status may be suggestive of high LR recurrence rates. In such cases, adjuvant RT may improve outcomes. When adjuvant RT is performed, the treatment volume should be well-designed so as to encompass the tumor bed, as well as LN stations No. 8, No. 9, No. 12, No. 14, and No. 16.

**Electronic supplementary material:**

The online version of this article (10.1186/s13014-018-1024-z) contains supplementary material, which is available to authorized users.

## Background

In developed countries, bile duct cancers are rare. Although the prevalence rates are gradually increasing, bile duct cancer is only the sixth most commonly occurring alimentary tract cancer in the United States of America [1]. However, higher incidence rates have been reported in northeast Thailand, and other Asian countries such as China and Korea [2, 3].

Bile duct cancers can be classified into 3 groups according to their locations: intrahepatic, perihilar, and distal bile duct cancers [4]. Most bile duct cancers occur in the perihilar bile duct (approximately 65–70%) followed by in the distal bile duct, which includes the ampulla of Vater (AOV) (approximately 25–30%) [2]. Intrahepatic bile duct cancer is relatively uncommon, accounting for approximately 5–10% of all bile duct cancers, and is associated with lower rates of lymph node (LN) metastases than cancers of the extrahepatic bile duct (EHBD) [2]. The EHBD (including the perihilar, and distal bile ducts, and the AOV) has a rich lymphatic network along the submucosa, and similar pathways of lymph drainage.

Surgical resection is the only cure for EHBD cancer patients, but the rates of resectability are not very high [5]. In addition, the 5-year survival rates after resection were still found to be poor, at approximately 10–40%, due to the high rates of loco-regional (LR) recurrence and distant metastasis (DM) [6]. Theoretically, when a patient has a high probability of recurrence, adjuvant therapy may be beneficial in improving outcomes. Nowadays, radiotherapy (RT) is often considered as adjuvant treatment in clinics when patients have positive resection margins (RMs) or pathologic LN metastases [7, 8]. However, there is a lack of randomized trials which focus on establishing which group of patients benefit from adjuvant RT; in addition, there are no definite guidelines in terms of proper RT target volumes.

The purpose of this study was to analyze the patterns of failure, especially LR failure, after surgical resection of EHBD cancer, and the prognostic factors for LR recurrence. This information can be used to guide the indication and treatment volumes for adjuvant RT.

## Methods

### Patient selection and data collection

Patients who underwent curative-intent resection of EHBD cancer, between 2001 and 2016, at 2 medical centers (Gyeongsang National University Hospital and Samsung Changwon Hospital), were selected for this retrospective analysis. The inclusion criteria were as follows: 1) presence of histologically proven bile duct cancer, 2) absence of gross residual disease after gross total resection, 3) absence of adjuvant RT use, and 4) presence of imaging studies to check the first site of recurrence. Patients who received adjuvant chemotherapy, and had microscopic residual disease were included in this analysis. In contrast, patients who had any of following conditions were excluded: 1) DM, 2) early death due to peri-operative complications, and 3) a previous history of other malignant diseases. We excluded 1 patient who had initial DM, 4 patients who died due to peri-operative complications, 1 patient with a history of other malignant disease, 2 patients who underwent adjuvant RT, and 1 patient who was diagnosed with high-grade dysplasia. As a result, a total of 93 EHBD cancer patients were eligible for this analysis. This study was approved by the Institutional Review Boards (IRBs) of Gyeongsang National University Hospital (IRB No. GNUH 2017–12-015) and Samsung Changwon Hospital (IRB No. SCMC 2017–05-007).

The images, surgical and histopathological records of all the patients were reviewed. Tumor location, presence of tumor satellite, surgical techniques, number of dissected LN, histology, pathologic stage (based on the seventh edition of the America Joint Committee on Cancer), RM status, and presence of and other adverse factors, such as lymphovascular invasion (LVI), perineural invasion (PNI) were recorded. Tumor location was categorized as perihilar bile duct (involving the bifurcation of the hepatic duct), distal bile duct (between the perihilar bile duct and the AOV), and the AOV (involving the union of the pancreatic duct and common bile duct). The post-surgical RM status was classified into 3 categories: R0, close, and R1. Close RM was defined as a distance of < 0.5 cm from the safety margin.

### Follow-up and statistical analysis

After surgery, patients were generally followed-up at 1 month, and then every 3 months for the first 2 years, followed by every 6 months for the next 3 years, with physical examinations and laboratory assessments including the measurement of the tumor markers. Abdominal and chest computed tomography (CT) was scanned every 6 months during the first 3 years, and annually during the next 2 years. Positron emission tomography (PET)-CT or magnetic resonance imaging were taken when recurrence was not clearly identified. All imaging studies were reviewed by a diagnostic radiologist, and the first site of recurrence was categorized as either local, regional, or distant failure. Local failure was defined as recurrence in the tumor bed or anastomosis site. Regional failure was defined as recurrence in the LR LN basin, as categorized by the Japanese Society of Hepato-Biliary-Pancreatic Surgery (Additional file 1) [9]. Distant failure was defined as recurrence outside of these areas.

Survival analyses were performed for overall and disease-free survival using the Kaplan-Meier method and log-rank test. Univariable and multivariable analyses were performed to predict the factors related to recurrence. The first sites of LR recurrence of each patient, identified in the follow-up images were plotted in an abdominal CT data set using Eclipse treatment planning system Version 13.7 (Varian Inc., Sunnyvale, CA, USA) and proposed by digitally reconstructed coronal radiograph images. All analyses were performed using SPSS software (Version 21.0; SPSS, Inc., Chicago, IL, USA). A two-sided *p* value < 0.05 was considered statistically significant.

## Results

### Patients

A total of 93 patients were included in this study. Sixty-two (66.7%) patients were treated at Gyeongsang National University Hospital, and 31 (33.3%) patients were treated at Samsung Changwon Hospital. The patients’ characteristics are shown in Table 1. Their median age at the time of surgery was 68 years (range, 51–80 years). Fifty-six (60.2%) patients were male. The numbers of patients in whom the location was the perihilar bile duct, distal bile duct, and AOV were 25 (26.9%), 52 (55.9%), and 16 (17.2%), respectively. Tumor satellites were found on initial work-up images of 2 (2.2%) perihilar bile duct cancer patients, and all of them underwent radical resection. Regarding surgical techniques, all patients underwent surgical resection with curative intent. For perihilar bile duct tumors, liver lobectomy with bile duct resection (17 patients) or bile duct resection with hepaticojejunostomy (8 patients) was conducted. For distal bile duct and AOV tumors, pancreaticoduodenectomy (29 patients), pylorus-preserving pancreaticoduodenectomy (35 patients), or bile duct resection (4 patients) was conducted. Regional LN dissection was performed in most patients (91 patients, 97.8%) and the median number of dissected LNs was 8 (range, 0–53). Thirty (32.3%) patients had pathologically positive LN metastasis, 32 (34.4%) patients had LVI, and 46 (49.5%) patients had PNI. The numbers of patients with R0, close, and R1 RMs were 59 (63.4%), 27 (29%), and 7 (7.5%), respectively. Most patients (*n* = 91, 97.8%) had adenocarcinoma, except for 1 patient who had adenosarcoma, and 1 patient who had carcinosarcoma. Adjuvant chemotherapy with fluoropyrimidine-based (8 patients) or gemcitabine-based (9 patients) regimens was administered to 17 (18.3%) patients.

**Table 1:** Patients’ characteristics

Characteristic		Number of patients (%)
Age (years)		Median: 68 (range, 51–80)
Sex	Male	56 (60.2)
	Female	37 (39.8)
Location	Perihilar bile duct	25 (26.9)
	Distal bile duct	52 (55.9)
	Ampulla of Vater	16 (17.2)
Tumor satellites	Present	2 (2.2)
	Absent	91 (97.8)
Pathologic T stage	T1–2	55 (59.1)
	T3–4	38 (40.9)
No. of LN dissection	≤8	50 (53.8)
	> 8	43 (46.2)
Pathologic N stage	N0	63 (67.7)
	N+	30 (32.3)
Histology	Adenocarcinoma	91 (97.8)
	Others	2 (2.2)
Histologic differentiation	WD-MD	74 (79.6)
	PD	19 (20.4)
Lymphovascular invasion	Yes	32 (34.4)
	No	61 (65.6)
Perineural invasion	Yes	46 (49.5)
	No	47 (50.5)
Resection margin status	R0	59 (63.4)
	Close RM-R1	34 (36.6)
Adjuvant chemotherapy	Fluoropyrimidine based	8 (8.6)
	Gemcitabine based	9 (9.7)
	No	76 (81.7)

*LN* lymph node, *N0* negative lymph node, *N+* positive lymph node, *WD* well differentiation, *MD* moderate differentiation, *PD* poor differentiation, *RM* resection margin

The median follow-up duration was 30 months (range, 4–147 months). Of the 93 patients, 41 (44.1%) died and 54 (58.1%) experienced a recurrence during the follow-up period. The 1-year, and 2-year overall survival rates were 90.6%, and 65.4%, respectively. 1-year, and 2-year disease-free survival rates were 62.9%, and 45.5%, respectively.

### Patterns of recurrence

**Fig. 1:**
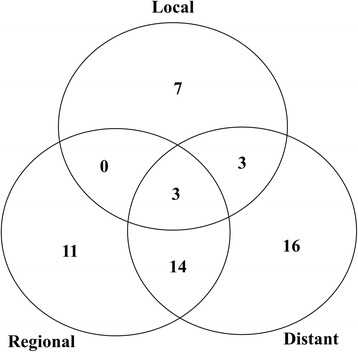
Overall patterns of failure

**Fig. 2:**
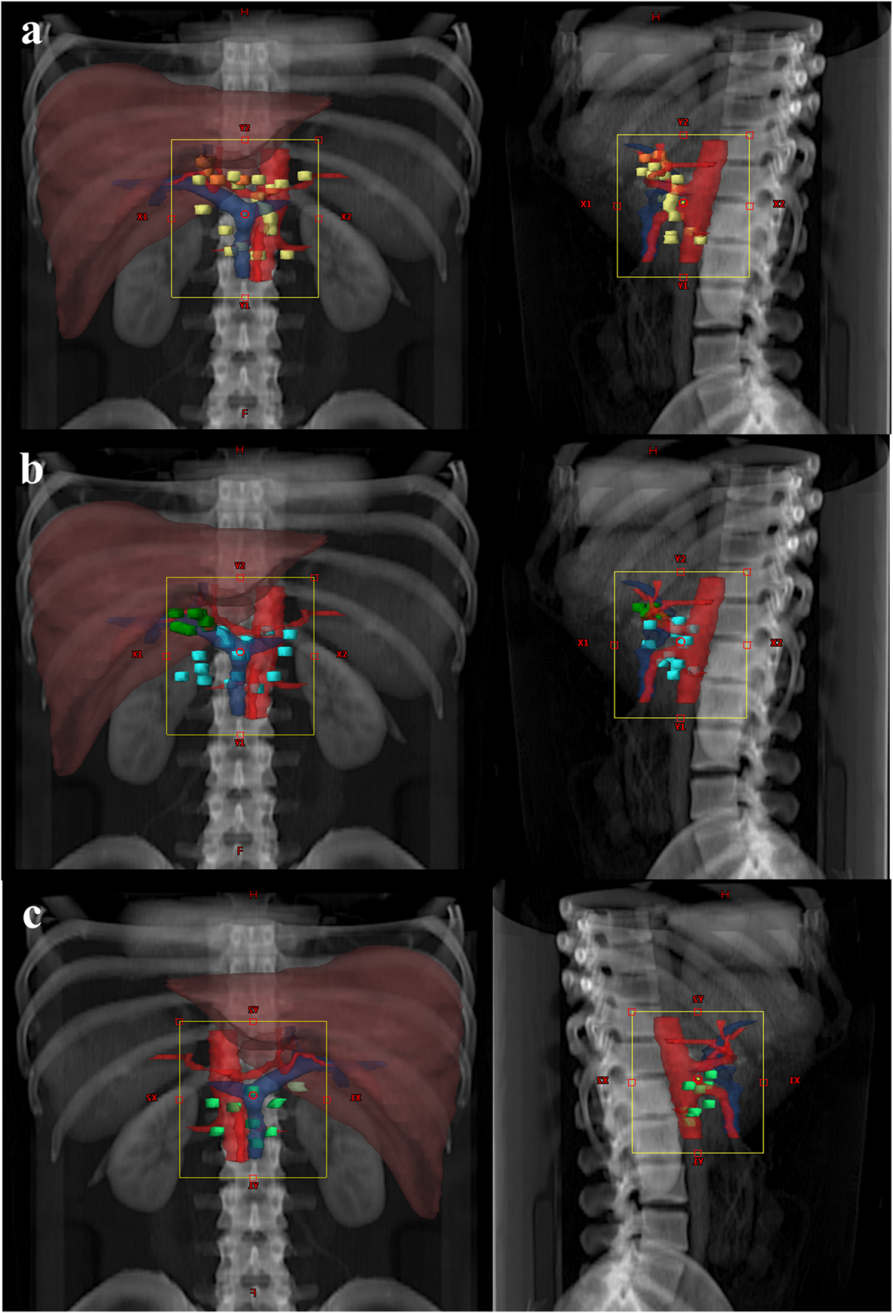
Sites of loco-regional recurrence according to tumor location. **a** Perihilar bile duct cancer. Abdominal aorta (red), portal vein (blue), local recurrence (orange), regional recurrence (yellow). **b** Distal bile duct cancer. Abdominal aorta (red), portal vein (blue), local recurrence (green), regional recurrence (cyan). **c** Ampulla of Vater cancer. Abdominal aorta (red), portal vein (blue), local recurrence (grass green), regional recurrence (light green)

Overall, 54 patients experienced recurrence. Of these patients, isolated-LR recurrence occurred in 18 (19.4%), and LR recurrence with concomitant DM occurred in 20 (21.5%) patients. DM with or without concomitant LR recurrence was observed in 36 (38.7%) patients; otherwise isolated DM was observed in 16 (17.2%) patients. These overall patterns of failure are shown in Fig. 1. Detailed data on the location of local recurrence and the LN station of regional recurrence, based on the Japanese classification, are shown in Tables 2, 3 and Fig. 2. Twenty-one LR recurrences were observed in 12 of 25 patients with perihilar bile duct cancer. The most frequent sites of failure were the tumor bed and LN stations No. 9 (*n* = 5, equally), followed by the LN stations No. 8 and 14 (*n* = 3, equally). In terms of distal bile duct cancer, 41 LR recurrences were observed in 22 of 51 patients. The most frequent site of failure was LN station No. 14 (*n* = 8), followed by the tumor bed and LN station No. 16 (*n* = 7, equally), and LN stations No. 8 and 12 (*n* = 6, equally). Nine LR recurrences occurred in 4 of 16 patients with cancers of the AOV. The most frequent site of failure was LN station No. 14 (*n* = 4), followed by LN station No. 16 (*n* = 2). LN station No. 16 was subdivided into 16a1, 16a2, 16b1, and 16b2. Of the 10 patients with LN station No. 16 metastases, the proportions of those with 16a1, 16a2, 16b1, and 16b2 were 1 (1.4%), 8 (11.3%), 1 (1.4%), and 0 (0%), respectively. Of the 36 patients with DM, the most common sites were the liver (*n* = 17), followed by peritoneal seeding (*n* = 11), and the lung (*n* = 6).

**Table 2:** Patterns of loco-regional recurrence based on the primary tumor location. The rate of loco-regional recurrence (%)

Site	LRR	TB	8 N	9 N	12 N	13 N	14N	16 N	17 N
PHBD	21	5	3	5	2	2	3	1	0
		(23.8)	(14.3)	(23.8)	(9.5)	(9.5)	(14.3)	(4.8)	(0)
DBD	41	7	6	4	6	2	8	7	1
		(17.1)	(14.6)	(9.8)	(14.6)	(4.9)	(19.5)	(17.1)	(2.4)
AOV	9	1	1	0	1	0	4	2	0
		(11.1)	(11.1)	(0)	(11.1)	(0)	(44.4)	(22.2)	(0)
Total	71	13	10	9	9	4	15	10	1
		(18.3)	(14.1)	(12.7)	(12.7)	(5.6)	(21.1)	(14.1)	(1.4)

*LRR* loco-regional recurrence, *TB* tumor bed, *N* lymph node station, *PHBD* perihilar bile duct, *DBD* distal bile duct, *AOV* ampulla of Vater

**Table 3:** Patterns of loco-regional recurrence based on the primary tumor location. The number of isolated lymph node station No. 16 failures (%)

Total	16 N	16a1	16a2	16b1	16b2
71	10	1	8	1	0
	(14.1)	(1.4)	(11.3)	(1.4)	(0)

*N*, lymph node station

### Prognostic factors for recurrence

In the univariable analysis, pathologically positive LN (*p* < 0.001), histology (*p* = 0.003), histologic differentiation (*p* = 0.012), LVI (*p* = 0.045), PNI (*p* = 0.001), and RM status (*p* < 0.001) were significant factors in predicting any type of recurrence. Pathologically positive LN (*p* = 0.002) and RM status (*p* = 0.001) remained significant in the multivariable analysis. Regarding LR recurrence, logistic regression analysis was performed to identify the potential indication of adjuvant RT. Only RM status (odds ratio, 2.382; 95% confidential interval 1.416–4.243; *p* < 0.001) was statistically significant in both the univariable and multivariable analyses. These results are shown in Table 4.

**Table 4:** Prognostic factors for (a) any type of recurrence and (b) loco-regional recurrence

(a) Any type of recurrence			
Univariable analysis			
Characteristics	OR	95% CI	*p* value
N0 vs N+	3.241	1.191–4.567	< 0.001
Others vs AD	0.102	0.023–0.456	0.003
WD-MD vs PD	2.164	1.185–3.951	0.012
LVI Yes vs No	0.566	0.325–0.987	0.045
PNI Yes vs No	0.385	0.219–0.677	0.001
R0 vs close RM-R1	3.351	1.923–5.841	< 0.001
Multivariable analysis			
Characteristics	OR	95% CI	*p* value
N0 vs N+	2.533	1.420–4.518	0.002
R0 vs close RM-R1	2.685	1.517–4.753	0.001
(b) loco-regional recurrence			
Univariable analysis			
Characteristics	OR	95% CI	*p* value
R0 vs close RM-R1	3.124	1.933–5.442	< 0.001
Multivariable analysis			
Characteristics	OR	95% CI	*p* value
R0 vs close RM-R1	2.382	1.416–4.243	< 0.001

*OR* odds ratio, *CI* confidential interval, vs versus, *N0* negative lymph node, *N+* positive lymph node, *AD* adenocarcinoma, *WD* well differentiation, *MD* moderate differentiation, *PD* poor differentiation, *LVI* lymphovascular invasion, *PNI* perineural invasion, *RM* resection margin

## Discussion

EHBD cancer has high rates of LR recurrence, even in patients who have undergone radical resection. These poor outcomes suggest that there is a requirement for adjuvant RT, with or without chemotherapy. However, due to the rarity in the occurrence of EHBD cancer, the role of adjuvant RT is poorly proven. Our study was implemented to evaluate the prognostic factors for recurrence and patterns of failure, particularly those associated with LR recurrence in patients with EHBD cancer who underwent surgical resection without adjuvant RT.

The role of adjuvant therapy in the treatment of EHBD cancer is poorly defined. Sikora et al. [10] reported that adjuvant chemoradiotherapy did not improve the survival or decrease the recurrence rates in AOV cancer patients who had undergone surgical resection. In another analysis of perihilar cholangiocarcinoma, performed by Henry et al. [11], it was reported that adjuvant RT did not prolong survival after radical resection. In contrast, several studies suggested that adjuvant therapy might be beneficial. Michael et al. [12] evaluated the outcome of adjuvant chemoradiotherapy after surgery in 34 patients with distal bile duct cancer, and found that adjuvant therapy improved local control and survival. Robert et al. [13] showed that adjuvant chemoradiotherapy may improve overall survival after resection, in a study of 125 AOV cancer patients. Recently, Kim et al. [14] reported the long-term outcomes of distal cholangiocarcinoma after surgery followed by adjuvant chemoradiotherapy; They reported slightly better outcomes in terms of 5-year LR-recurrence-free survival, disease-free survival, and overall survival rates (70.7%, 49.4%, and 48.1%, respectively) compared to other surgery alone studies. The above-mentioned studies showing improved outcomes reported that poor histologic differentiation, LN metastasis, positive RM status, and a higher primary tumor stage were the main factors behind LR recurrence; therefore, the use of adjuvant therapy targeted at these factors may reduce the recurrence rates. A systematic review and meta-analysis by Jennifer et al. [7] studied the benefit of adjuvant therapy for biliary tract cancer. They reported that the use of adjuvant therapy provided the greatest benefit in biliary tract cancer patients with an LN positive status (odds ratio, 0.49; *p* = 0.004) and R1 status (odds ratio, 0.36; *p* = 0.002). In our study, we observed an LR recurrence rate of 40.9%, which is higher than the reported rate of 25.8% (Kim et al.) after resection followed by adjuvant chemoradiotherapy [14]; this difference suggests the role of adjuvant therapy in LR recurrence. The multivariable analysis, performed to predict any type of recurrence, showed that positive LN metastasis and RM status were significant factors. Regarding the prediction of LR recurrence, in the univariable and multivariable analysis, RM status remained significant. Therefore, we suggest that the addition of adjuvant therapy may improve outcomes in patients with positive LN metastases and RM status; in particular, in patients with a positive RM status, adjuvant RT may improve LR control.

DM is also a major pattern of failure in patients with EHBD cancer who have undergone resection. Several studies have attributed the inefficiency of adjuvant therapy to the fact that DM accounts for a large portion of recurrence [15]. Kim et al. [14] reported that DM is still a major pattern of failure for EHBD cancer patients who have undergone adjuvant chemoradiotherapy, and the pattern seems to shift from LR recurrence to DM. In our study, DM occurred in 38.7% of the patients and LR recurrence was observed in 40.9%. Concomitant LR recurrence and DM was observed in 20 (21.5%) patients. To control DM, further advances in chemotherapy are needed, which focus on defining when chemotherapy should be used, and the identification of novel agents.

Recently, similar to our study, a retrospective study about LR recurrence patterns and prognostic factors in patients with cholangiocarcinoma who underwent surgical resection without RT was reported by Ghiassi-Nejad et al. [16]. Their study included a total of 145 patients and median followed up 41.6 months, and reported recurrence in 59% of patients (LR recurrence, 51%; DM, 16.3%). In addition, they reported the presence of satellite nodule and stage 3/any LN status were significant prognostic factors of overall recurrence. Compared with our results, a similar rate of any type of recurrence event was observed, but the detailed LR recurrence and DM were somewhat different. These differences may be due to differences in the proportion of patients with intrahepatic tumors (0% vs. 70.3%), who did not received LN dissection (2.2% vs. 31%) or who underwent adjuvant chemotherapy (18.3% vs. 26%).

Currently, the treatment volumes for adjuvant RT are poorly defined. In the modern era, three-dimensional conformal RT, intensity-modulated RT, or stereotactic radiosurgery are conducted in hepatobiliary cancer patients for reduced treatment-related toxicity, while maintaining the quality of treatment [17, 18]. With changes in RT, precise target delineation has become more important. Traditionally, the RT target volume for EHBD cancers in adjuvant setting varied, and roughly included the tumor bed and LN basin (porta hepatis, hepatic artery, pancreaticoduodenal, celiac, and superior mesenteric artery lymph node). Several studies [10, 12, 13, 15, 18–21] have focused on adjuvant RT in EHBD cancer patients, and their RT volumes are shown in Table 5. Sikora et al. [10] conducted adjuvant RT in AOV cancer patients including the tumor bed and porta hepatis. Another study by Robert et al. [13] which implemented RT included the tumor bed, porta hepatis, peripancreatic, superior mesenteric, and para-aortic nodes from approximately the T11/12 to L2/3 vertebral body levels. In our study, the first site of LR recurrence was analyzed. Our results suggest that the tumor bed and the following LN stations should be included in the RT target volume, due to the associated high rate of LR recurrence: LN stations No. 8 (frequency = 14.1%), No. 9 (12.7%), No. 12 (12.7%), No. 14 (21.1%), and No. 16 (14.1%). With regards to LN station No. 16, 16a2 (frequency = 11.3%) should be included in the treatment volume, but 16a1 (1.4%), 16b1 (1.4%), and 16b2 (0%) may be omitted for reduced RT-related toxicity. As shown in Fig. 2, while the patterns were similar for the different primary tumor locations, we observed a gradual downward trend, by tumor location, from the perihilar bile duct to the AOV. For the coverage of these areas, we suggest the superior border at the T10/11 interspace to cover the tumor bed, inferior border at the L1/2 interspace to cover the proper paraaortic LN adjacent to the renal hilum, and lateral border 1 cm from the vertebra body to cover the left lateralized metastatic paraaortic LN in the anteroposterior/posteroanterior RT field. With this sophisticated target setting, dose escalation through the intensity-modulated RT technique may be possible to overcome the poor treatment outcomes.

**Table 5:** Studies on adjuvant radiotherapy in extrahepatic bile duct cancer cases and their treatment volumes

Study	Location	Adjuvant RT volume
Robert et al. [13]	AOV	TB + porta hepatis, PP, celiac, SM, PA (T11/12 – L2/3) nodes
Sikora et al. [10]	AOV	TB + porta hepatis nodes
Brian et al. [15]	EHBD	TB + porta hepatis, pericholedochal, PD, celiac nodes
Ha et al. [19]	EHBD	TB + porta hepatis, PD, celiac nodes
Michael et al. [12]	DBD	TB + primary node stations + PA (T10/11 – L2/3) nodes
Takeshi et al. [20]	PH	TB + porta hepatis, hepatic artery nodes
Ben-Josef et al. [21]	EHBD	TB + porta hepatis, retroPD, celiac nodes
Our study	EHBD	TB + LN station of No. 8, 9, 12, 14, and 16a2

*RT* radiotherapy, *AOV* ampulla of Vater, *EHBD* extrahepatic bile duct, *DBD* distal bile duct, *PH* perihilar, *TB* tumor bed, *PP* peripancreatic, *SM* superior mesenteric, *PA* paraaortic, *PD* pancreaticoduodenal, *LN* lymph node

There are several limitations to this study. First, its retrospective nature may have influenced the heterogeneous patient characteristics, especially the small number of patients who received adjuvant chemotherapy and the relatively small number of cancer patients. Second, the criteria for the judgment of recurrence by a diagnostic radiologist, and surgical technique used by a general surgeon were not constant, as this study was performed in 2 medical centers. Future multicenter randomized trials with a large number of patients are needed to confirm the role of adjuvant RT and definite RT target volume in preventing recurrence.

## Conclusions

In conclusion, our results suggest that a close or R1 RM status may be predictive of high LR recurrence rates. For patients with these risk factors, adjuvant RT may be required. If adjuvant RT is conducted, the treatment field should encompass the tumor bed, and LN stations No. 8, No. 9, No. 12, No. 14, and No. 16 in order for the frequently affected LR recurrence areas to be covered.

## Additional file

Additional file 1: Location and number of abdominal lymph node stations. (TIF 1122 kb)

## Abbreviations

AOV: Ampulla of vater
CT: Computed tomography
DM: Distant metastasis
EHBD: Extrahepatic bile duct
IRB: Institutional review board
LN: Lymph node
LR: Loco-regional
LVI: Lymphovascular invasion
PNI: Perineural invasion
RM: Resection margin
RT: Radiotherapy
