# Supplementary figures and images for: Patterns of failure after resection of extrahepatic bile duct cancer: implications for adjuvant radiotherapy indication and treatment volumes
Source: Radiat Oncol. 2018 May 8;13:85. doi: 10.1186/s13014-018-1024-z (PMC5941763; doi:10.1186/s13014-018-1024-z)

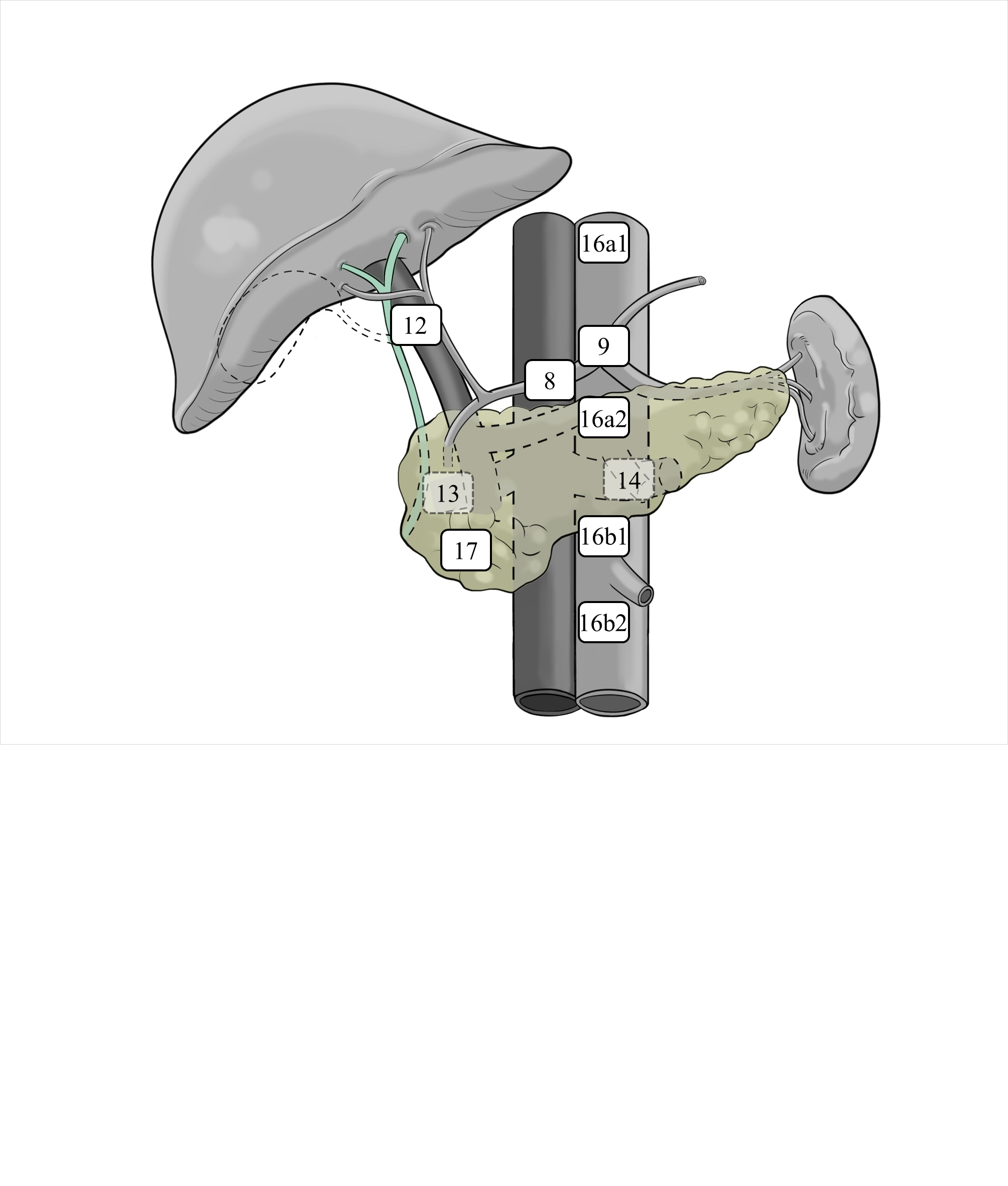

Supplement: Supplementary file 1 — Location and number of abdominal lymph node stations. (TIF 1122 kb) [file 13014_2018_1024_MOESM1_ESM.tif]
